# Incidence, Nature and Natural History of Additional Histological Findings in Preimplantation and Implantation Kidney Transplant Biopsies

**DOI:** 10.3389/ti.2024.12997

**Published:** 2024-08-14

**Authors:** A. L. Paterson, V. Broecker, M. Gray, A. Chalisey, G. J. Pettigrew, D. M. Summers

**Affiliations:** ^1^ Department of Histopathology, Cambridge University Hospitals NHS Foundation Trust, Cambridge, United Kingdom; ^2^ Department of Clinical Pathology, Sahlgrenska University Hospital, Gothenburg, Sweden; ^3^ Department of Transplant Surgery, Cambridge University Hospitals NHS Foundation Trust, Cambridge, United Kingdom; ^4^ Department of Medicine, Cambridge University Hospitals NHS Foundation Trust, Cambridge, United Kingdom; ^5^ Department of Surgery, University of Cambridge, Cambridge, United Kingdom

**Keywords:** kidney transplantation, preimplantation biopsy, histopathology, donor utilisation, implantation biopsy

## Abstract

The quality assurance provided by preimplantation biopsy quantification of chronic damage may allow greater use of kidneys from expanded criteria donors, and thereby expand the deceased donor pool. Preimplantation biopsy may, however, identify additional acute or chronic pathologies not considered in the scoring of chronic damage, and these may influence the decision to implant or discard the kidney. This single-centre retrospective cohort study of a contemporary UK donor population systematically characterised the nature of additional findings in 1,046 preimplantation and implantation biopsies over an eight-year period. A diverse range of findings were identified in 111/1,046 (11%) organs; most frequently diabetic glomerulopathy, focal segmental glomerulosclerosis, (micro)thrombi, neutrophil casts, and immunoglobulin/complement staining. Seventy (63%) of these were transplanted, with subsequent biopsy in 41 (58%) cases confirming that 80% of the initial acute changes had spontaneously resolved, while there was no progression of diabetic glomerulopathy, and the lesions of focal segmental glomerulosclerosis were not identified. Over 75% of assessable grafts with additional histological findings at the time of transplant showed adequate function at one-year following transplant. In conclusion, most histological abnormalities that may be identified in addition to chronic scarring in preimplantation kidney biopsies would not preclude transplantation nor predict poor graft function.

## Graphical Abstract

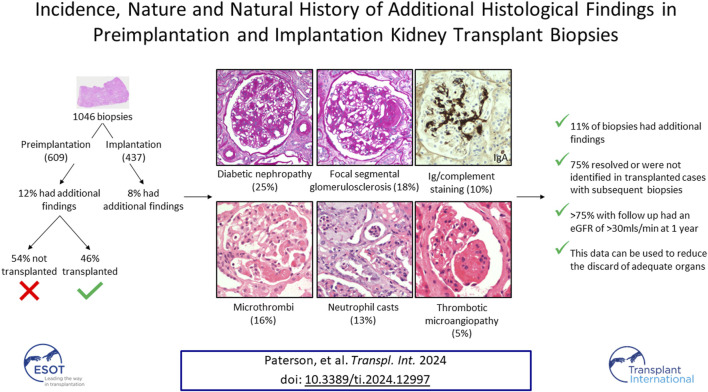

## Introduction

Kidney transplantation is the most cost-effective treatment for end-stage kidney disease and can improve both survival and quality of life for most patients when compared to dialysis [1]. In 2021-2022 just over 2000 kidney-only deceased donor transplants were undertaken in the United Kingdom (UK) [2]. Despite this, in March 2023 there remained over 5,500 adult patients active on the kidney transplant waiting list demonstrating the significant mismatch between the number of organs being transplanted and those required [2]. Multiple strategies are being explored and adopted to increase both organ donation and utilisation in the UK, including changes to the law regarding consent for deceased donation, increasing donation after circulatory death (DCD), and considering single or dual kidney transplants from donors of older age and/or donors with co-morbidities that previously would have led to them being considered unsuitable [3–7].

There is a substantially increased risk of graft loss following transplantation of kidneys from older donors and/or those with significant comorbidities. Along with consideration of donor and recipient characteristics and macroscopic evaluation of the donor kidney, histological assessment of the donor kidney may be undertaken following retrieval to inform the decision regarding organ quality [8, 9]. Preimplantation biopsy assessment is routinely available to the Transplant team at our centre and uses a scoring system based on the Remuzzi score [9]. The Pre-Implantation Trial of Histopathology In renal transplant Allografts (PITHIA trial) was undertaken at the other twenty-one kidney transplant centres in the UK in 2019–2022, with the aim of determining whether access to preimplantation kidney biopsies at a national level would increase the number and quality of organs being implanted [10]. The results are awaited. In cases where a preimplantation biopsy is not taken, a biopsy taken at the time of kidney reperfusion and assessed in a similar manner can provide a useful baseline assessment of organ quality and has been shown to correlate with allograft survival even after adjusting for donor characteristics [11, 12].

The Remuzzi score assesses four parameters associated with chronic damage: global glomerulosclerosis; tubular atrophy; interstitial fibrosis; and arteriosclerosis. Additional histological abnormalities that are not considered for this score may, however, also be revealed. These may impact on the decision to transplant the organ and on peri-transplant management of the recipient. The available literature describing additional histological findings in either preimplantation kidney biopsies or those taken at organ implantation, almost exclusively focuses on the significance of either glomerular microthrombi or incidental IgA deposits. Glomerular fibrin thrombi have been described in 3%–10% of preimplantation and/or implantation kidney biopsies [13–15], particularly in donors with a history of central nervous system injury secondary to trauma [13, 14, 16, 17]. In most cases the fibrin thrombi were focal, rapidly resolved and were not associated with adverse long-term outcomes [13–16]. IgA deposits have been reported in 9%–29% of peri-transplant kidney biopsies, with a higher prevalence in donors of Hispanic or Asian origin [18–20]. The majority were of a low intensity and without associated hypercellularity, consistent with latent IgA deposition, and cleared on follow up biopsies [18–20].

Importantly, the nature of additional histological findings will be influenced by the characteristics of the donor pool and therefore differ between countries and over time as organ utilisation practice evolves. The majority of the literature is based on cohorts from the United States, whereas kidney donors in the UK are: significantly older (with 11% being ≥70 years old); more frequently DCD donors; less likely to have died from trauma; and a higher proportion are Caucasian [21].

This study aimed to facilitate future informed decision-making regarding organ use and recipient management by systematically reviewing the frequency and spectrum of additional histological findings in transplant biopsies taken prior to, or at the time of, transplantation from a contemporary UK donor population. The natural history of these changes was explored in biopsies taken after transplantation during standard practice follow up to determine whether the observed abnormalities persisted, progressed or resolved.

## Patients and Methods

This was a retrospective single-centre cohort study. The local electronic record was searched to identify all kidney transplant biopsies taken either prior to transplantation (preimplantation), or following reperfusion (implantation), from 1st November 2014 until 31st December 2022. Preimplantation biopsies were taken at the discretion of the on-call consultant transplant surgeon and consultant nephrologist based on the donor’s history and macroscopic appearances of the organ. Circumstances where a preimplantation biopsy would be considered included advanced donor age, a donor history of long-standing hypertension and/or being on multiple antihypertensive medications for hypertension control, a donor history or finding of cardiovascular disease such as left ventricular hypertrophy which may suggest co-existing renovascular disease, a donor history of diabetes mellitus, an abnormal donor creatinine without a baseline preceding the current illness, atherosclerosis in the arterial patch, and/or macroscopic features suggestive of cortical scarring. Implantation biopsies were taken at the discretion of the consultant surgeon following reperfusion, particularly in older donors. All the biopsies were reported by subspecialist renal pathologists at the study centre. The study was registered and approved as a service evaluation at our institution (clinical project ID5034); ethical approval was not required in accordance with the local legislation and institutional requirements.

Preimplantation biopsies, typically 4 mm punch biopsies, were formalin-fixed, underwent rapid processing, and were then paraffin-embedded. A ten-slide serial was cut with two profiles per slide. Slides 1, 5, and 10 were stained with haematoxylin and eosin. Slides 2, 6, and 9 were stained with periodic acid Schiff (PAS). The biopsies were assessed using the Remuzzi score [9] and other pertinent findings noted. The findings were then discussed between the reporting pathologist and on-call transplant surgeon. Implantation biopsies were formalin-fixed, paraffin-embedded and cut as per preimplantation biopsies, however they had a longer processing time and were reported within normal working hours as the biopsy result would not influence immediate decisions regarding transplantation.

The histopathology reports for all preimplantation and implantation biopsies were reviewed to identify those with findings in addition to those captured as part of the Remuzzi score, other than acute tubular injury. For preimplantation biopsies, the donor organ donation and transplantation number was cross-checked with the NHS Blood and Transplant (NHSBT) register to determine which kidneys were subsequently transplanted. Further biopsies taken following transplant were identified from the local electronic record and the reports reviewed to determine whether the changes identified at preimplantation or implantation had persisted. These were indication biopsies, rather than protocol biopsies, that were taken to investigate delayed graft function or an unexplained rise in serum creatinine. Demographic and clinical follow up data for the transplanted cases was accessed from a combination of the local electronic records and the NHSBT register depending on the location of follow up after transplantation. Twelve month estimated glomerular filtration rate (eGFR) was used as the clinical outcome measure. Urine albumin-creatinine ratio (uACR) was used to assess the degree of proteinuria following transplantation; <30 mg/mmol was considered to be mild, 30–70 mg/mmol moderate; and >70 mg/mmol severe. The final timepoint of data collection was in December 2023, 13 months after the last transplant.

## Results

A total of 1,046 biopsies were assessed during the study time-period, comprising 609 preimplantation biopsies from 404 donors, and 437 implantation biopsies. Significant histological findings in addition to global glomerulosclerosis, tubular atrophy, interstitial fibrosis and/or vascular sclerosis were identified in 76 (12%) preimplantation biopsies from 50 donors, and 35 (8%) implantation biopsies from 34 donors [total 111 additional pathologies in 1,046 (11%) biopsies]. Donor demographic data is shown in Supplementary Table S1. The nature of the additional abnormalities was diverse (see Table 1), but with diabetic glomerulopathy (25%), focal segmental glomerulosclerosis (FSGS; 18%), and deposition of thrombi/microthrombi (16%), reported most frequently (Table 1). The diabetic changes varied in severity, with approximately half showing predominantly pre-nodular glomerulopathy, and the remainder, nodular glomerulopathy.

**TABLE 1:** Nature of the additional histological findings identified in preimplantation biopsies where the organ was or was not subsequently used; and in implantation biopsies. *Three cases had two co-existing additional findings; ^ one case had two co-existing additional findings; ^+^one case had three co-existing additional findings and one case two co-existing additional findings.

Finding	Preimplantation not transplanted (n = 41*)	Preimplantation transplanted (n = 35^**∧**^)	Implantation (n = 35^+^)	All biopsies with additional findings (n = 111)
**Glomerular Changes**				
Diabetic glomerulopathy – all Prenodular Nodular	15/41 (37%) 8/41 (20%) 7/41 (17%)	11/35 (31%) 5/35 (14%) 6/35 (17%)	2/35 (6%) 2/35 (6%) -	28/111 (25%) 15/111 (14%) 13/111 (12%)
Focal segmental glomerulosclerosis	13/41 (32%)	4/35 (11%)	3/35 (9%)	20/111 (18%)
Thrombi/microthrombi – all Focal Diffuse	--- --- ---	6/35 (17%) 4/35 (11%) 2/35 (6%)	12/35 (34%) 8/35 (23%) 4/35 (11%)	18/111 (16%) 12/111 (11%) 6/111 (5%)
Complement/immunoglobulin staining	---	4/35 (11%)	7/35 (20%)	11/111 (10%)
Glomerulonephritis	2/41 (5%)	---	2/35 (6%)	4/111 (4%)
Hyperfiltration features	2/41 (5%)	2/35 (6%)	---	4/111 (4%)
**Vascular Changes**				
Thrombotic microangiopathy	2/41 (5%)	2/35 (6%)	1/35 (3%)	5/111 (5%)
Infarction	2/41 (5%)	---	1/35 (3%)	3/111 (3%)
Focal arteriolar necrosis	1/41 (2%)			1/111 (1%)
Cholesterol emboli	---	1/35 (3%)	1/35 (3%)	2/111 (2%)
Arteriolar hyalinosis	---	---	1/35 (3%)	1/111 (1%)
**Tubulointerstitial Changes**				
Neutrophil casts	7/41 (17%)	2/35 (6%)	5/35 (14%)	14/111 (13%)
Obstructive features	---	2/35 (6%)	---	2/111 (2%)
Myoglobin casts	---	2/35 (6%)	---	2/111 (2%)
Interstitial foam cells	---	---	1/35 (3%)	1/111 (1%)
Interstitial calcium deposits	---	---	1/35 (3%)	1/111 (1%)
Tubulointerstitial inflammation	---	---	1/35 (3%)	1/111 (1%)

Forty-one (54%) kidneys with significant additional findings in the preimplantation biopsy were not subsequently transplanted (Tables 1, 2), with the most frequent findings being diabetic glomerulopathy (37%), FSGS (32%) and/or neutrophil casts suggestive of ascending infection (17%). Sixteen (39%) of these biopsies had a Remuzzi score of 4 or less, and therefore the kidney could have been considered for single transplantation, with the additional findings likely a factor in the organ discard. Similarly, a further fifteen (37%) had a Remuzzi score of 5 or 6, and could have been considered for dual transplant if both kidneys had been locally available [10], with the additional histopathological findings potentially contributing to the decision not to implant. Seven (17%) had a Remuzzi score of 7 or more and would therefore not have been considered suitable for transplantation regardless of the additional histological findings. In the remaining three cases the biopsy sample was insufficient for Remuzzi scoring.

**TABLE 2:** Additional findings in preimplantation biopsies which were not subsequently used shown by Remuzzi score group. ^ one case had both neutrophil casts and FSGS, *two cases had both nodular diabetes and FSGS.

	Remuzzi score 0–4 (n = 16^∧^)	Remuzzi score 5-6 (n = 15)	Remuzzi score 7–12 (n = 7*)	Insufficient for Remuzzi scoring (n = 3)
**Glomerular Changes**				
Diabetic glomerulopathy - all Prenodular Nodular	8/16 (50%) 4/16 (25%) 4/16 (25%)	3/15 (20%) 2/15 (13%) 1/15 (7%)	4/7 (57%) 2/7 (29%) 2/7 (29%)	--- --- ---
Focal segmental glomerulosclerosis	2/16 (13%)	8/15 (53%)	3/7 (43%)	---
Glomerulonephritis	2/16 (13%)	---	---	---
Hyperfiltration features	---	2/15 (13%)	---	---
**Vascular Changes**				
Thrombotic microangiopathy	---	---	1/7 (14%)	1/3 (33%)
Infarction	2/16 (13%)			
Focal arteriolar necrosis	---	1/15 (7%)	---	---
**Tubulointerstitial Changes**				
Neutrophil casts	3/16 (19%)	1/15 (7%)	1/7 (14%)	2/3 (67%)

Additional preimplantation or implantation histological findings were identified in 70 kidneys which were transplanted into 67 recipients during the study time-period. Recipient demographic data is shown in Supplementary Table S2. The most prevalent abnormalities were: the presence of (micro)thrombi (26%); diabetic glomerulosclerosis (19%); complement and/or immunoglobulin deposition (16%); FSGS (10%); and neutrophils casts 10%. Data regarding follow up biopsies were available for 59/67 (88%) of these recipients. The remaining organs were transplanted at other UK centres. The number of biopsies following transplant ranged from 1–5 biopsies per recipient (median 2 biopsies), with 37/59 (63%) having at least one subsequent biopsy during follow up. Of these, 24% were taken in the first 10 days after transplant, 69% within the first 6 months and 81% within the first year. The findings from this cohort are summarised in Table 3.

**TABLE 3:** Proportion of transplanted kidneys with additional histological abnormalities in preimplantation or implantation biopsies which had further biopsies following transplant. The number where the peri-transplant change had resolved or was not identified in the latest biopsy following transplant is shown.

Finding	Proportion with follow up biopsies	Preimplantation/implantation change resolved or not identified
**Glomerular Changes**		
Diabetic glomerulopathy – all Prenodular Nodular	6/13 (46%) 4/7 (57%) 2/6 (33%)	3/6 (50%) 3/4 (75%) 0/2 (0%)
Focal segmental glomerulosclerosis	5/7 (71%)	5/5 (100%)
Thrombi/microthrombi – all Focal Diffuse	12/18 (67%) 9/12 (75%) 3/6 (50%)	9/12 (75%) 8/9 (89%) 1/3 (33%)
Complement/immunoglobulin staining	5/11 (45%)	4/5 (80%)
Glomerulonephritis	2/2 (100%)	1/2 (50%)
Hyperfiltration features	2/2 (100%)	1/2 (50%)
**Vascular Changes**		
Thrombotic microangiopathy	2/3 (67%)	2/2 (100%)
Infarction	0/1 (0%)	---
Cholesterol emboli	1/2 (50%)	1/1 (100%)
Arteriolar hyalinosis	0/1 (0%)	---
**Tubulointerstitial Changes**		
Neutrophil casts	3/7 (43%)	3/3 (100%)
Obstructive features	1/2 (50%)	0/1 (0%)
Myoglobin casts	1/2 (50%)	1/1 (100%)
Interstitial foam cells	0/1 (0%)	---
Interstitial calcium deposits	0/1 (0%)	---
Tubulointerstitial inflammation	0/1 (0%)	---

In those with a follow up biopsy, in 8 of 9 (89%) with focal microvascular thrombi, this had resolved. In the remaining case where a small arterial thrombus was present in the implantation biopsy, a subsequent biopsy on day 29 revealed an organised infarct. Despite this, the kidney transplant continues to provide good function at 5 years following transplant, with a stable eGFR of 45 mL/min. Two patients with more widespread microvascular thrombi at implantation, who had received a kidney from the same DCD donor, had a diffuse severe ischaemic injury comprising areas of infarction on subsequent biopsies taken on days 7, 8, and 36. Both had a subsequent transplant 6 months later due to on-going poor graft function. In the remaining patient with widespread microvascular thrombi at baseline and a follow up biopsy, the changes had resolved by day 7.

In 4 of 5 (80%) patients with complement/immunoglobulin deposition present on preimplantation or implantation biopsy, these deposits had resolved on follow-up biopsy. In one patient IgA deposition was still identified on biopsy at day 124 after transplant. Eight patients with complement/immunoglobulin deposition on the preimplantation or implantation biopsy had follow-up at our centre, none had a uACR more than 10 mg/mmol on samples taken within the first 3 months following transplantation, median uACR 5 mg/mmol (range 1–10 mg/mmol).

In three patients with neutrophil casts identified on preimplantation/implantation biopsy, these were no longer evident on subsequent biopsy taken on days 5, 6, and 140 after transplant. One patient without a follow up biopsy had a positive urine culture for *Proteus* species within the first 30 days following transplant; they had received a dual transplant and preimplantation biopsies from both kidneys had shown the presence of focal neutrophil casts. This patient was successfully treated with antibiotics and had an eGFR of 35 mL/min 1 year following transplant. Small numbers of preimplantation or implantation biopsies revealed features of urinary obstruction, glomerulonephritis without positive staining for complement/immunoglobulin, or glomerular hypertrophy, with persisting changes observed in one case each on repeat biopsy on day 7, day 82, and day 1919 following transplant, respectively (Table 3).

Seven preimplantation/implantation biopsies revealed FSGS; the number of lesions were small with only one (4/7, 57%) or two lesions (3/7, 43%) of segmental sclerosis per case, with a median of 23 glomeruli, range 8–59, on the assessed level per case. Further lesions of FSGS were not present in the 10 biopsies performed on 5 of these kidneys after transplantation, consistent with the FSGS lesions only involving a small proportion of glomeruli. This is supported by all five cases with follow up at our centre having a uACR less than 30 mg/mmol following transplantation consistent with at most mild proteinuria.

Follow up biopsies were available in 6 of 13 patients who had diabetic glomerulopathy identified in the preimplantation/implantation biopsy (Table 3). In three patients, the diabetic glomerular changes were not reported on their latest biopsies (performed on days 6, 218 and 1,428 after transplantation), suggesting that diabetic features had either resolved or were not prominent. In the remaining three, the diabetic changes were still present in the most recent follow up biopsy (on days 328, 427, and 447) and although numbers are small, the features that persisted were typically the more advanced nodular forms of glomerulopathy (Table 3). Arteriolar hyalinosis, another feature of diabetes-related kidney disease was noted in one patient at day 6 following transplant.

One year graft eGFR was available for 63/67 (94%) patients who had received a graft with additional histological findings on preimplantation/implantation biopsy. One patient died with a functioning graft 7 months after transplant following cardiac surgery, whilst data was not available for three patients who had received their transplant at other UK centres. These four cases have been removed from subsequent analyses. At 1 year, 48/63 (76%) of patients had an eGFR >30 mL/min consistent with adequate graft function, Table 4. All assessable patients with neutrophil casts, focal microthrombi or hypercellular glomeruli at the time of transplantation had adequate graft function at 1 year. Four (66%) of those with diffuse glomerular microthrombi had adequate function at 1 year; the remaining two patients as detailed above received a kidney from the same DCD donor with subsequent poor function, despite preimplantation Remuzzi scores of 0 and 1 (Supplementary Table S3).

**TABLE 4:** Proportion of recipients receiving grafts with additional histological findings at baseline who had a 3-month and/or 12-month estimated glomerular filtration rate (eGFR) greater that 30 mL/min. ^+^Three patients received a dual transplant and are shown once. The data is shown per additional finding at the time of transplant, one graft had two co-existing abnormalities and one graft had three co-existing abnormalities. *one and ^two had unknown function at 12 months; ^#^one patient died 7 months following transplant from an unrelated cause–all these patients have been removed from the respective columns including one with dual pathology.

	Proportion of recipients with eGFR at 3 months >30 mL/min	Proportion of recipients with eGFR at 12 months >30 mL/min
**Glomerular Changes**		
Diabetic glomerulopathy – all Prenodular Nodular	10/11 (91%)^^^ 6/6 (100%)* 4/5 (80%)*	9/12 (75%)* 6/7 (86%) 3/5 (60%)*
Focal segmental glomerulosclerosis	3/6 (50%)*	3/5 (60%)^
Thrombi/microthrombi - all Focal Diffuse	15/18 (83%) 11/12 (92%) 4/6 (66%)	16/18 (89%) 12/12 (100%) 4/6 (67%)
Complement/Immunoglobulin staining^+^	8/9 (89%)*	7/9 (78%)*
Glomerulonephritis	1/2 (50%)	2/2 (100%)
Hyperfiltration features	0/2 (0%)	0/2 (0%)
**Vascular Changes**		
Thrombotic microangiopathy	1/3 (33%)	0/3 (0%)
Infarction	1/1 (100%)	1/1 (100%)
Cholesterol emboli	2/2 (100%)	1/2 (50%)
Arteriolar hyalinosis	1/1 (100%)	1/1 (100%)
**Tubulointerstitial Changes**		
Neutrophil casts^+^	5/6 (83%)	5/5 (100%)^#^
Obstructive features^+^	1/1 (100%)	1/1 (100%)
Myoglobin casts	2/2 (100%)	2/2 (100%)
Interstitial foam cells	1/1 (100%)	1/1 (100%)
Interstitial calcium deposits	1/1 (100%)	1/1 (100%)
Tubulointerstitial inflammation	1/1 (100%)	1/1 (100%)

The majority [9/12 (75%)] of kidneys transplanted with features of diabetic glomerulopathy at baseline biopsy achieved an eGFR >30 mL/min at 1 year, with a higher proportion of those with early, prenodular features doing so than those with more established nodular features, albeit numbers are small in each group (Table 4). The Remuzzi score did not add value when predicting which organs with nodular diabetic glomerulosclerosis would have adequate subsequent function (Supplementary Table S3). None of the six patient with prenodular diabetic glomerulopathy that were transplanted at our centre had more than mild proteinuria on a uACR taken within the first 3 months, median 10 mg/mmol (range 5–29 mg/mmol). Four of the six patients with nodular diabetic glomerulopathy had uACR measurements available following transplantation. In two patients these showed moderate proteinuria at 3 months, further measurements were available for one of these patients which showed a reduction in proteinuria to 14 mg/mmol at 10 months following transplant. Two patients had severe proteinuria 3 months following transplantation, 71 mg/mmol and 118 mg/mmol, which had increased to 120 mg/mmol and 584 mg/mmol respectively 12 months following transplantation although in the latter case the increase is likely partly attributable to the development of chronic active antibody mediated rejection which also resulted in a fall in eGFR from 62 mL/min at 3 months to 22 mL/min at 12 months.

With regards to FSGS on baseline biopsy, 3/5 (60%) kidney grafts had an eGFR >30 mL/min at 1 year. The two organs with poor function both had a Remuzzi score of 4, which was higher than the grafts which had an adequate subsequent function (Supplementary Table S1), suggesting that the combination of a higher Remuzzi score and FSGS may be a marker of potential poor function however the study is not powered for further statistical analysis.

None of the three kidneys with features of thrombotic microangiopathy (TMA) at the time of transplant had adequate function at 1 year; although in two cases the TMA had resolved on biopsies taken on day 9 and 16 following transplant. In the first case, features of TMA were identified in the preimplantation biopsy in one glomerulus and postulated to be related to peri-transplant ischaemia. Subsequent biopsies for delayed graft function showed severe acute tubular injury with parenchymal necrosis. The persistent poor function was considered most likely to reflect donor vascular disease and interstitial fibrosis. The implantation biopsy for the second patient that showed TMA also received a Remuzzi score of 5, indicating moderately severe chronic injury, which may explain the persistent suboptimal function. The third kidney achieved excellent early graft function however was removed at 2 months due to haemorrhage from nephrostomy placement, which had been indicated because of the development of ureteric stenosis.

Both kidneys with glomerular hypertrophy at baseline had poor function from the time of transplant and an eGFR <30 mL/min at 1 year following transplant, although with 12-month uACRs of 6 mg/mmol and 7 mg/mmol consistent with minimal proteinuria. They were from the same donor who had a history of severe left ventricular dysfunction, congestive cardiac failure and hypertension however had an eGFR >60 mL/min 8 days prior to organ donation and 37 mL/min at the time of donation. Preimplantation biopsies showed diffuse glomerular enlargement with mild vascular disease although without global glomerulosclerosis and with only focal tubular atrophy and interstitial fibrosis affecting less than 5% of the cortical area, both Remuzzi score 3, which would predict adequate function following transplantation. The poor graft function therefore could not have been anticipated and is likely specific to the circumstances of this donor.

## Discussion

This retrospective study of a large UK donor population has demonstrated that, in addition to the features considered for Remuzzi evaluation, preimplantation biopsy reveals histological abnormalities in up to 12% of biopsies. A broad range of abnormalities were identified; some anticipated, such as diabetic glomerulosclerosis; and others unanticipated, such as glomerular microthrombi or intratubular neutrophil casts. Forty-one organs with additional histological findings on preimplantation biopsies were not transplanted, of which 16 (39%) and 15 (37%) would have otherwise been considered for single or dual organ transplantation respectively based on the Remuzzi score. This suggests that the additional findings may have contributed to discard/decline of up to an extra 5% of kidneys that were biopsied. On follow up of transplanted organs with these findings, the majority of acute changes resolved after transplantation, and even chronic features were not always identified in subsequent biopsies, presumably because they were focal and non-progressive within the timeframe of the study. Furthermore, in the majority the 12-month eGFR achieved was >30 mL/min. This suggests that these histological features may have led to the unnecessary discard of some otherwise transplantable organs.

The most commonly observed additional feature identified on preimplantation/implantation biopsy was diabetic glomerulosclerosis, present in 2.7% of biopsies. The prevalence of diabetes mellitus in deceased donors in the UK is approximately 7%, increasing to 9% in those ≥60 years old [6, 21]. There is, however, limited literature on the prevalence, natural history and function of grafts with diabetic glomerulosclerosis at baseline. Truong et al [22] reported that baseline biopsy revealed diabetic glomerulopathy in only 19% of donors with a history of diabetes, and that all were at an early stage, with only a minority progressing following transplantation; with similar findings in a subsequent study of thirty-four recipients [23]. Similarly, in the time course of our study, no increase in disease stage was identified. This also provides reassurance that the preimplantation/implantation biopsy did not underestimate the stage of disease. All but one graft with prenodular diabetic glomerulosclerosis at the time of transplant achieved adequate function 12 months later, although this was only true for 60% of donors with nodular diabetic glomerulosclerosis. This data would suggest that adequate function, at least in the short term, would be anticipated when utilising organs with prenodular diabetic glomerulosclerosis and these are therefore suitable for use; however caution is required if nodular diabetic glomerulosclerosis is identified.

Focal and diffuse microvascular thrombi were identified in 1.1% and 0.5% of all biopsies respectively. Microthrombi were more prevalent in implantation than preimplantation biopsies, as observed previously, which may partly reflect reperfusion injury [13, 15]. In keeping with previous studies [13, 14, 16], most follow up biopsies revealed no sequelae, apart from two patients whose clinical course likely reflects peri-transplant events, including a prolonged warm ischaemia time, specific to their shared donor. This highlights the importance of integrating biopsy results with the broader clinical context. The study data, when combined with that in the literature, supports the use of kidneys for transplantation where the preimplantation biopsy has shown focal microvascular thrombi; however if diffuse thrombi are present a more detailed consideration in combination with other case-specific factors such as warm and cold ischaemic time and Remuzzi score would be appropriate as occasional cases have poor outcomes post transplantation.

Intratubular neutrophil casts were identified in 1.3% of biopsies. Their presence, particularly when accompanied by neutrophilic tubulitis and surrounding inflammation, or in biopsies from older and/or diabetic patients, may suggest an ascending urinary tract infection [24, 25]. However the feature is not specific, occurring in other causes of acute tubular injury and interstitial inflammation [26], and it is not possible to judge the overall extent of inflammation. Hence when neutrophil casts are isolated and focal, their significance is uncertain. All transplanted organs with this finding had adequate function at 12 months, apart from one recipient who died from an unrelated cause with a functioning graft. One recipient had a potentially donor-derived urinary tract infection which was successfully treated. These observations suggest that focal neutrophil casts on a preimplantation biopsy should not automatically result in organ discard. However if used, transport medium cultures, urine cultures and/or prophylactic antibiotics should be considered.

Immunoglobulin and/or complement deposition, most frequently IgA, was identified in 1.1% of biopsies, with minimal hypercellularity and resolution in most cases with a follow-up biopsy. Latent IgA deposits have long been recognised at the time of transplantation, particularly in Asian and Hispanic donors, with the majority resolving rapidly and spontaneously without negatively impacting on long term function [18, 19]. Knowledge of these deposits is most relevant when interpreting biopsies following transplantation but would not influence immediate decisions regarding transplantation, because immunohistochemistry analysis is not possible while maintaining acceptably short cold ischaemic times. Given the good outcomes of these transplanted organs both in this study and the literature, minor features on a preimplantation biopsy such as slight mesangial expansion and/or hypercellularity which may suggest IgA nephropathy in the donor, should not preclude organ use. There is insufficient data to draw conclusions regarding the use of organs with endocapillary hypercellularity.

The remaining additional findings were diverse and, apart from FSGS, were each restricted to an occasional donor. The clinical relevance of some, such as features of obstruction, glomerulomegaly or interstitial foam cells is unclear, particularly if isolated findings. Similarly with other features, such as cholesterol thromboemboli, it is difficult to know from the single biopsy if these are focal or represent a more widespread embolic shower that may lead to subsequent scarring. Where follow up biopsies were available, few changes persisted or progressed. Therefore a discussion between the Histopathologist and Transplant team regarding the nature and extent of additional findings, their likely significance, and any potential sequelae in addition to the Remuzzi score is suggested.

Despite our study covering over 8 years and a thousand biopsies, due to the diversity of the histological findings, each subcohort contains a small number of transplanted organs which limits conclusions regarding graft outcome. However over 75% of transplanted organs had a 12-month eGFR of >30 mL/min consistent with adequate graft function [27]. Exploration of the natural history of the histological changes is further impacted by only a subset having further biopsies and the majority occurring within the first year following transplant. This is most relevant for grafts with chronic changes since progression or even regression of structural changes is likely to occur over long timeframes. Our study cohort included both preimplantation and implantation biopsies in order to maximise the size of these subgroups and data available following transplant. Whilst the incidence of the different additional histological findings does vary between the two groups, in part due to their different demographics, the natural history of the additional pathological findings following transplantation would be expected to be similar.

In summary, a diverse range of histological findings may be encountered in preimplantation/implantation biopsies from a UK donor population. Most acute changes are anticipated to spontaneously resolve without sequelae, whilst most chronic changes appear to be focal, at an early stage and/or non-progressive. This study suggests that many of these organs are suitable for transplantation and would be anticipated to have adequate function at 1 year. In the context of an organ shortage this would further expand the donor pool.

## Data Availability

The datasets presented in this article are not readily available because it comprises medical information related to study patients. Requests to access the datasets should be directed to alp37@cam.ac.uk.

## Abbreviations

DCD, donation after circulatory death; NHSBT, National Health Service Blood and Transplant; eGFR, estimated glomerular filtration rate; uACR, urine albumin-creatinine ratio; FSGS, focal segmental glomerulosclerosis; TMA, thrombotic microangiopathy.
