# Supplementary material for: Incidence, Nature and Natural History of Additional Histological Findings in Preimplantation and Implantation Kidney Transplant Biopsies
Source: Transpl Int. 2024 Aug 14;37:12997. doi: 10.3389/ti.2024.12997 (PMC11349550; doi:10.3389/ti.2024.12997)
Supplement: Supplementary file 1 [file Table1.docx]

Supplementary Table 1: Demographic characteristics of donors where additional histological findings were found in the preimplantation or implantation biopsy. The single live donor has been removed from the implantation group.

|  | Preimplantation biopsies (n=50) | Implantation  biopsies (n=33) |  |
| --- | --- | --- | --- |
| Age (median (interquartile range)) | 59 (50-67) | 52 (46-58) | p=0.020 |
| Male  Female | 33 (66%)  17 (34%) | 20 (61%)  13 (39%) | p=0.617 |
| History of diabetes | 21 (42%) | 4 (12%) | p=0.004 |
| History of hypertension | 26 (52%) | 10 (30%) | p=0.051 |
| Pre-donation creatinine umol/L  (median (interquartile range)) | 90 (72-142) | 50 (72-111) | P=0.015 |
