# Supplementary material for: Incidence, Nature and Natural History of Additional Histological Findings in Preimplantation and Implantation Kidney Transplant Biopsies
Source: Transpl Int. 2024 Aug 14;37:12997. doi: 10.3389/ti.2024.12997 (PMC11349550; doi:10.3389/ti.2024.12997)
Supplement: Supplementary file 2 [file Table2.docx]

Supplementary Table 2: Demographics of recipients who received a donor organ with additional histological findings. *The single live donor has been removed from these categories. ^+^Calculated reaction frequency 0% unsensitised, 1-84% sensitised, 85%+ highly sensitised. ^Reference: M Marlais, A Hudson, L Pankhurst, SV Fuggle, SD Marks. Living donation has a greater impact on renal allograft survival than HLA matching in pediatric renal transplant recipients. Transplantation 2016; 100:2717-2722. ^#^Defined as Banff 1A acute T cell mediated rejection or higher and/or antibody mediated rejection.

|  |  | Recipients (n=67) |
| --- | --- | --- |
| Age | Median (interquartile range) | 54 (44-64) |
| Sex | Male | 39 (58.2%) |
|  | Female | 28 (41.8%) |
| Cause of renal failure | Hypertension/Renovascular Disease | 3 (4.5%) |
|  | Diabetes | 3 (4.5%) |
|  | Polycystic kidney disease | 9 (13.4%) |
|  | Pyelonephritis/Interstitial Nephritis | 5 (7.5%) |
|  | Glomerulonephritis | 16 (23.9%) |
|  | Miscellaneous | 20 (29.9%) |
|  | Not Reported | 11 (16.4%) |
| Dialysis status | Predialysis | 14 (20.9%) |
|  | Dialysis | 53 (79.1%) |
| Waiting time* | Median days (interquartile range) | 610 (238-963) |
| Diabetes | Yes | 11 (16.4%) |
|  | No | 49 (73.1%) |
|  | Unknown | 7 (10.4%) |
| Previous kidney transplant | Yes | 5 (7.5%) |
|  | No | 55 (82.1%) |
|  | Unknown | 7 (10.4%) |
| Sensitisation^+^ | Unsensitised | 44 (65.7%) |
|  | Sensitised | 14 (20.9%) |
|  | Highly sensitised | 9 (13.4%) |
| Donor | DBD | 30 (44.8%) |
|  | DCD | 36 (53.7%) |
|  | Live related | 1 (1.5%) |
| Cold ischaemic time* | Median hours (interquartile range) | 16.0 (13.4-19.0) |
| HLA matching^ | Level 1 | 1 (1.5%) |
|  | Level 2 | 10 (14.9%) |
|  | Level 3 | 42 (62.7%) |
|  | Level 4 | 14 (20.9%) |
| Delayed graft function | Yes | 22 (32.8%) |
|  | No | 42 (62.7%) |
|  | Unknown | 3 (4.5%) |
| Biopsy proven rejection in first 12 months^#^ | Yes | 6 (9.0%) |
|  | No | 54 (80.6%) |
|  | Unknown | 7 (10.4%) |
