# Supplementary material for: Incidence, Nature and Natural History of Additional Histological Findings in Preimplantation and Implantation Kidney Transplant Biopsies
Source: Transpl Int. 2024 Aug 14;37:12997. doi: 10.3389/ti.2024.12997 (PMC11349550; doi:10.3389/ti.2024.12997)
Supplement: Supplementary file 3 [file Table3.docx]

Supplementary table 3: Preimplantation/implantation Remuzzi scores and 12-month estimated glomerular filtration rate (eGFR). IA – inadequate for Remuzzi scoring. ^+^Three patient received a dual transplant and are shown once; their Remuzzi scores were 5-5, 5-5 and 4-4. The data is shown per additional finding at the time of transplant, ^&^one graft had two co-existing abnormalities and ^%^one graft had three co-existing abnormalities. *One and ^two had unknown function at 12 months; ^#^one patient died seven months following transplant from an unrelated cause – all four patients have been removed for this analysis including one with dual pathology.

|  | 12 month eGFR >30mls/min | | | | | | | 12 month eGFR <30mls/min | | | | | | |
| --- | --- | --- | --- | --- | --- | --- | --- | --- | --- | --- | --- | --- | --- | --- |
|  | Remuzzi score | | | | | | | | | | | | | |
|  | 0 | 1 | 2 | 3 | 4 | 5 | IA | 0 | 1 | 2 | 3 | 4 | 5 | IA |
| Diabetic glomerulopathy - all*  Prenodular  Nodular* | 1  1  - | 1  -  1 | 1  1  - | 2  2^%^  - | 3  2  1 | 1  -  1 | -  -  - | -  -  - | -  -  - | -  -  - | 1  -  1 | 1  -  1 | 1  1  - | -  -  - |
| Focal segmental glomerulosclerosis^ | - | - | 2 | - | - | - | 1 | - | - | - | - | 2 | - | - |
| Thrombi/microthrombi - all  Focal  Diffuse | 3  2  1 | 2  2  - | 5  4  1 | 1  -  1^%^ | 3  3  - | -  -  - | 2  1^&^  1 | 1  0  1 | 1  0  1 | -  -  - | -  -  - | -  -  - | -  -  - | -  -  - |
| Complement/Immunoglobulin staining* | 3 | - | 1 | 1^%^ | 1^+^ | - | 1^&^ | - | 1 | - | - | 1 | - | - |
| Glomerulonephritis | - | 1 | 1 | - | - | - | - | - | - | - | - | - | - | - |
| Hyperfiltration features | - | - | - | - | - | - |  | - | - | - | 2 | - | - | - |
| Thrombotic microangiopathy | - | - | - | - | - | - | - | - | - | 1 | - | - | 1 | 1 |
| Infarction | - | - | - | - | - | - | 1 | - | - | - | - | - | - | - |
| Cholesterol emboli | - | - | 1 | - | - | - | - | - | - | - | - | 1 | - | - |
| Arteriolar hyalinosis | 1 | - | - | - | - | - | - | - | - | - | - | - | - | - |
| Neutrophil casts^#^ | - | - | 1 | 1 | 2 | 1^+^ | - | - | - | - | - | - | - | - |
| Obstructive features | - | - | - | - | - | 1^+^ | - | - | - | - | - | - | - | - |
| Myoglobin casts | 2 | - | - | - | - | - | - | - | - | - | - | - | - | - |
| Interstitial foam cells | - | 1 | - | - | - | - | - | - | - | - | - | - | - | - |
| Interstitial calcium deposits | 1 | - | - | - | - | - | - | - | - | - | - | - | - | - |
| Tubulointerstitial inflammation | - | - | 1 | - | - | - | - | - | - | - | - | - | - | - |
| Total | 11 | 5 | 13 | 3 | 9 | 3 | 4 | 1 | 2 | 1 | 3 | 5 | 2 | 1 |
